# Genetic and Environmental Risk Factors for Intermittent Explosive Disorder, ADHD and Conduct Disorder: Shared and Unique Influences

**DOI:** 10.1002/cpp.70195

**Published:** 2025-12-15

**Authors:** Fangqing Liu

**Affiliations:** ^1^ The University of Manchester Manchester UK

**Keywords:** attention‐deficit hyperactivity disorder, conduct disorder, genetics, intermittent explosive disorder, risk factors

## Abstract

**Objective:**

This systematic review examines the shared and unique risk factors for intermittent explosive disorder (IED), attention‐deficit/hyperactivity disorder (ADHD) and conduct disorder (CD), aiming to provide a comprehensive understanding of their etiological pathways and inform future intervention strategies.

**Methods:**

We conducted a comprehensive search across seven databases (Web of Science, APA PsycINFO, MEDLINE, PubMed, Cochrane, CENTRAL and Embase) for studies examining risk factors for IED, ADHD and CD. A total of 44 studies were included, focusing on genetic, environmental and psychosocial factors. We employed the Preferred Reporting Items for Systematic Review and Meta‐Analysis (PRISMA) guidelines for study selection and quality assessment.

**Results:**

Forty‐four studies were included. We identified 15 cross‐disorder risk factors. Of these, nine domains had evidence across all three disorders. The remaining six domains showed a more restricted pattern (present for ADHD and/or CD, but not yet studied or not reported for IED): maternal smoking during pregnancy, maternal alcohol use during pregnancy, low birth weight/other perinatal risks, parental psychopathology/maternal mental health, MAOA/COMT genetic variants and parenting stress/school‐related disadvantage that has not been tested for IED.

**Conclusion:**

Our findings emphasise the importance of shared environmental and biological factors across these disorders, with implications for integrated intervention strategies. The review highlights gaps in research and calls for more targeted studies that incorporate gender, developmental stage and family dynamics.

## Summary

- Screen for shared adversities early. Children and adolescents presenting with ADHD‐, CD‐ or IED‐like behaviours should routinely be screened for family violence/maltreatment, low socioeconomic stressors and suboptimal/harsh parenting, because these were the most consistently shared risks across all three disorders.
- Use family‐ and school‐based supports as first‐line contexts. Because many risks operate through the caregiving environment and school failure/peer conflict, integrating parent‐management/parent‐training work with school behaviour supports is likely to benefit not only ADHD and CD cases but also youths with anger‐outburst/IED presentations.
- Adapt ADHD/CD interventions to IED profiles. Existing, well‐supported externalising‐disorder programmes (emotion coaching, de‐escalation in the moment, consistent routines) can be realistically ‘translated’ to IED, even though IED has been less studied, to help caregivers manage trigger‐bound anger episodes.
- Consider developmental timing and gender. Risk exposure (e.g., harsh parenting at ages 2–5 years, school problems in later childhood) and possible sex differences should guide when and for whom to intervene, rather than waiting for a full diagnostic picture to emerge.
- Watch for unmeasured prenatal/biological risks in IED. Prenatal/perinatal complications and inflammatory pathways are well described in ADHD/CD but underexamined in IED; clinicians working with IED should document these systematically to inform future integrated care pathways.

## 1 Introduction

Intermittent explosive disorder (IED) is characterised by sudden, excessive and recurrent angry outbursts triggered by minor daily events (Scott et al. 2020). The global prevalence of IED is estimated to be between 5.1% (lifetime) and 8.9% (12 months), depending on the time frame of prevalence (Liu and Yin 2025). Notably, there has been an upward trend in IED diagnoses over the past 10 years (Liu and Yin 2024), which may be attributable to recent changes in the DSM‐5‐Text Revision (TR), including verbal aggression in its criteria. While behaviour problems associated with IED may diminish with age (Coccaro 2012), research has shown that adolescents with IED, if left untreated, are at an increased risk of experiencing poor social relationships, academic underperformance (Samek and Hicks 2014) and criminal involvement (Cook et al. 2010; Meehan et al. 2017; Young et al. 2020).

**FIGURE 1:**
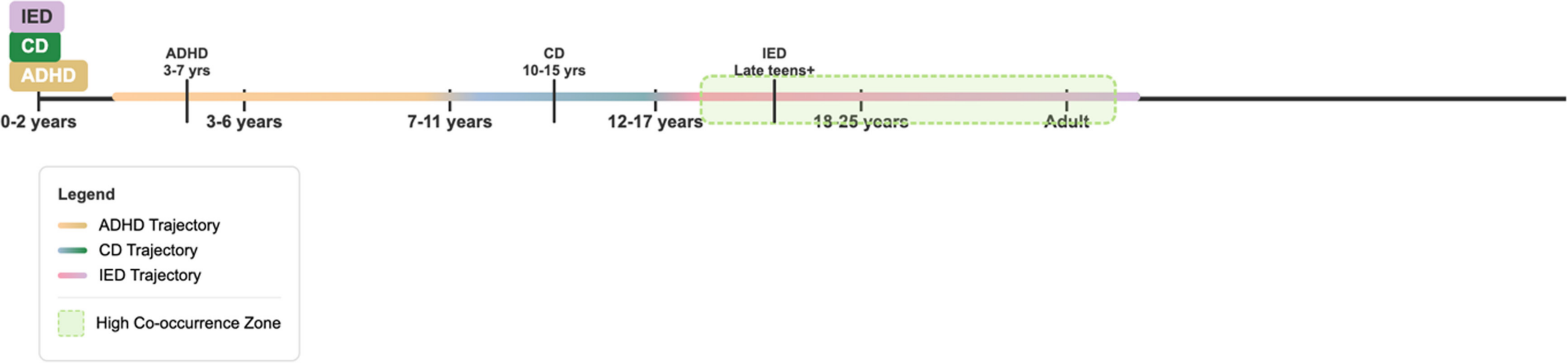
Developmental trajectories and high co‐occurrence zones of ADHD, CD and IED. The high co‐occurrence zone is shown within the shaded green area.

A growing body of research suggests that IED frequently co‐occurs with other psychiatric disorders, particularly attention‐deficit/hyperactivity disorder (ADHD) and conduct disorder (CD) (Barra et al. 2022; Gnanavel et al. 2019). The lifetime comorbidity rate of ADHD and IED ranges from 9.1% (Radwan and Coccaro 2020) to 36% (Coccaro 2012). Similarly, IED and CD frequently co‐occur, often following distinct developmental trajectories. ADHD typically manifests in early childhood, while CD tends to emerge in adolescence and may precede the onset of IED. Among adults, CD and IED appear to develop at similar rates, though their sequence of emergence varies across individuals (Barra et al. 2022; see Figure 1).

These distinct developmental patterns point to the existence of shared underlying risk factors and reinforce the need for an integrated approach to understanding these disorders. Despite these common origins and overlapping features, such as emotional dysregulation, poor impulse control and interpersonal difficulties, IED, ADHD and CD are also characterised by distinct symptomatology (Ogundele 2018; Savolainen et al. 2015). For instance, ADHD is primarily associated with sustained attention difficulties that IED does not typically exhibit. While both IED and ADHD can involve impulsive actions, IED specifically entails episodes of severe, explosive anger, which are not typical of ADHD. Similarly, CD and IED both involve aggressive behaviours, but the aggression in CD is typically more calculated and premeditated, in contrast to the impulsive, anger‐driven outbursts commonly seen in IED (Ogundele 2018; Savolainen et al. 2015).

Several theoretical models have been proposed to explain the high rates of comorbidity among mental health disorders (McGrath et al. 2020). One of the most widely recognised models is the correlated risk factors model, which posits that comorbidity arises from shared biological and environmental risk factors (Harvey et al. 2016). Evidence from twin studies has robustly supported the genetic basis of this overlap (Eaves et al. 2000; Tuvblad et al. 2009), showing that common heritable factors contribute to the co‐occurrence of ADHD and CD. Beyond genetics, shared environmental influences, such as exposure to early‐life adversity, family conflict and socio‐economic stress, also play an important role in shaping these disorders (Burt et al. 2001). Given this complex interplay, it is reasonable to hypothesise that both biological predispositions and environmental factors explain the covariation among ADHD, IED and CD symptoms. However, the research of risk factors for CD and ADHD is relatively more advanced than for IED (Abdelnour et al. 2022; Wesseldijk et al. 2018). In contrast, such comprehensive treatment paradigms for IED are still underdeveloped, highlighting the need for more in‐depth research to uncover its underlying mechanisms, risk factors, developmental trajectories and effective interventions (Radwan and Coccaro 2020).

A key methodological gap that this review addresses is the lack of comprehensive studies that directly compare the environmental, genetic and psychosocial risk factors across IED, ADHD and CD. While individual studies have examined the risk factors for each disorder in isolation, there has been no systematic attempt to integrate these findings and evaluate the overlapping and distinct risk factors across these conditions. Therefore, this review aims to address this gap by systematically comparing and synthesising the shared and unique risk factors for IED, ADHD and CD.

### 1.1 Research Objectives

The present review has three specific research objectives: (1) We synthesise evidence on putative antecedents and vulnerability factors that appear to be shared across IED, ADHD and CD, in order to clarify whether there is a common liability profile that cuts across these externalising phenotypes. (2) We examine candidate risk factors that are uniquely or preferentially reported for each disorder, with the aim of distinguishing disorder‐specific pathways. (3) We critically evaluate gaps in the current literature, including inconsistent diagnostic definitions, developmental timing of measurement and limited longitudinal data for IED, and outline priority directions for future research and early intervention.

## 2 Methods

**FIGURE 2:**
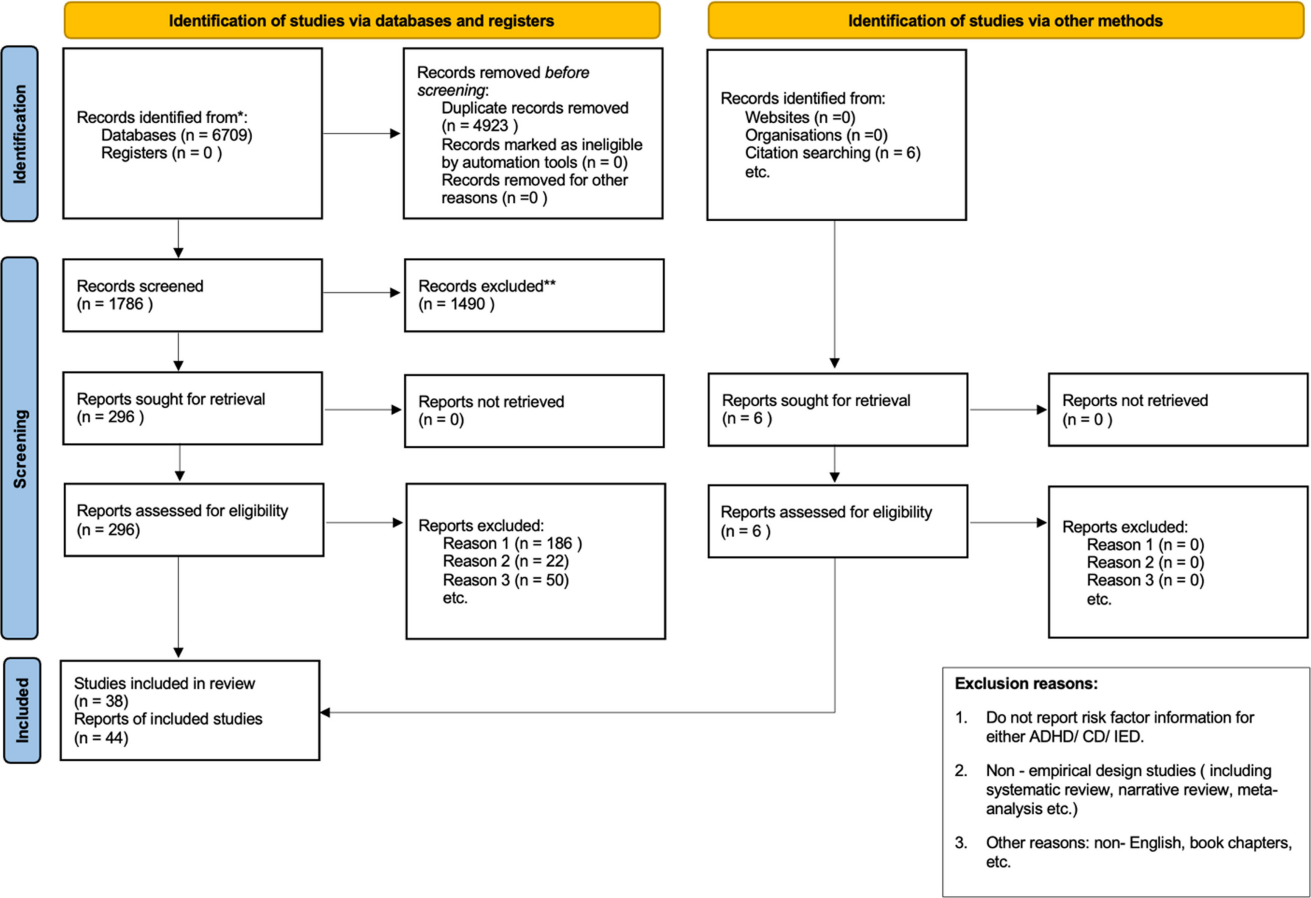
PRISMA flowchart for study selection and inclusion (Page et al. 2021).

This systematic review was reported following the Preferred Reporting Items for Systematic Review and Meta‐Analysis (Page et al. 2021), and this review is registered in the International Prospective Register of Systematic Reviews (registration ID: CRD42024553357). The completed PRISMA checklist is available in Appendix A, and the study selection flow is shown in the PRISMA diagram (Figure 2).

### 2.1 Search Strategy

In this systematic review, the articles were sourced from an extensive search across seven databases in August 2025: Web of Science, APA PsycINFO, MEDLINE with Full Text, PubMed, Cochrane, CENTRAL and Embase. Additionally, manual searches complemented this strategy as a secondary method to minimise reporting bias. We strategically combined keywords, including ‘attention deficit/hyperactivity disorder’ or ‘ADHD’, ‘conduct disorder’ or ‘CD’, ‘intermittent explosive disorder’, “impuls*’ or ‘IED’, along with ‘risk factor’, ‘predict*’ and ‘determinant’. Boolean operators ‘OR’ and ‘AND’ were employed to merge these terms, with specific syntax adjustments for each database to optimise the search. We also screened reference lists of included studies where available. The comprehensive search strategy used and a sample search results can be found in Appendix B.

The inclusion criteria for this systematic review required participants to have a diagnosis of ADHD, CD or IED based on either DSM or ICD criteria or to meet the clinical or research cut‐off points on a validated screening instrument. Only studies involving human participants were included. Nonempirical publications, such as case reports, book chapters, letters, editorials and opinion pieces, as well as studies employing nonexperimental designs, were excluded. In addition, studies for which the full text could not be retrieved were excluded to ensure consistency in data extraction and analysis. No restrictions were imposed on language or year of publication.

### 2.2 Study Selection

Our comprehensive search yielded 6709 records, with an additional six identified through manual reference screening. After duplicate removal using Coevidence, 1786 unique records remained for initial screening. Two independent reviewers, both with master's degrees in psychology and specialised expertise in mental health, screened all records. Discrepancies were resolved through discussion until consensus was reached. The screening process comprised two stages: an initial review of titles and abstracts (*κ* = 0.86), followed by full‐text assessment of potentially eligible studies (*κ* = 0.88). In total, 44 studies met the inclusion criteria and were retained for data extraction and analysis. The same reviewers also independently assessed the methodological quality of each included study.

### 2.3 Data Extraction

Data from each selected study were extracted and recorded in. The form includes information regarding (1) authors' names and publication year; (2) sample characteristics: detailed information about the study participants, including the sample size, age range, gender distribution, inclusion/exclusion criteria and any other relevant demographic characteristics; (3) study design: the type of study design employed, such as cross‐sectional, longitudinal, experimental or case–control; (4) disorders included in the study: a list of the specific disorders examined in the study, with particular attention to how the study operationalised and diagnosed each condition, including diagnostic criteria used (e.g., DSM‐5, ICD‐10); (5) study objectives: a summary of the primary aims and hypotheses of the study; and (6) study main findings: a concise description of the key results and conclusions drawn from the study, highlighting significant findings related to risk factors, correlations, treatment outcomes or other relevant data. This section also notes any limitations or noteworthy considerations that impact the interpretation of the results (see in Appendix C).

### 2.4 Quality Assessment

These 44 articles were evaluated using the NIH Quality Assessment Tool for Observational Cohort and Cross‐Sectional Studies (National Institutes of Health 2013), a 14‐item inventory used to evaluate quantitative and mixed‐method studies. Two independent reviewers completed the evaluation procedure and discrepancies raised were discussed, and a consensus was reached. The quality assessment results can be found in Appendix D.

### 2.5 Data Synthesis

Meta‐analysis was not conducted due to heterogeneity in diagnostic assessment, participant age groups, experimental paradigms and, thus, measurements of outcomes. Given these differences between studies, it was judged that it would not be valid to pool effect sizes across studies. Therefore, instead, a narrative synthesis approach was used to combine results across studies.

## 3 Results

**FIGURE 3:**
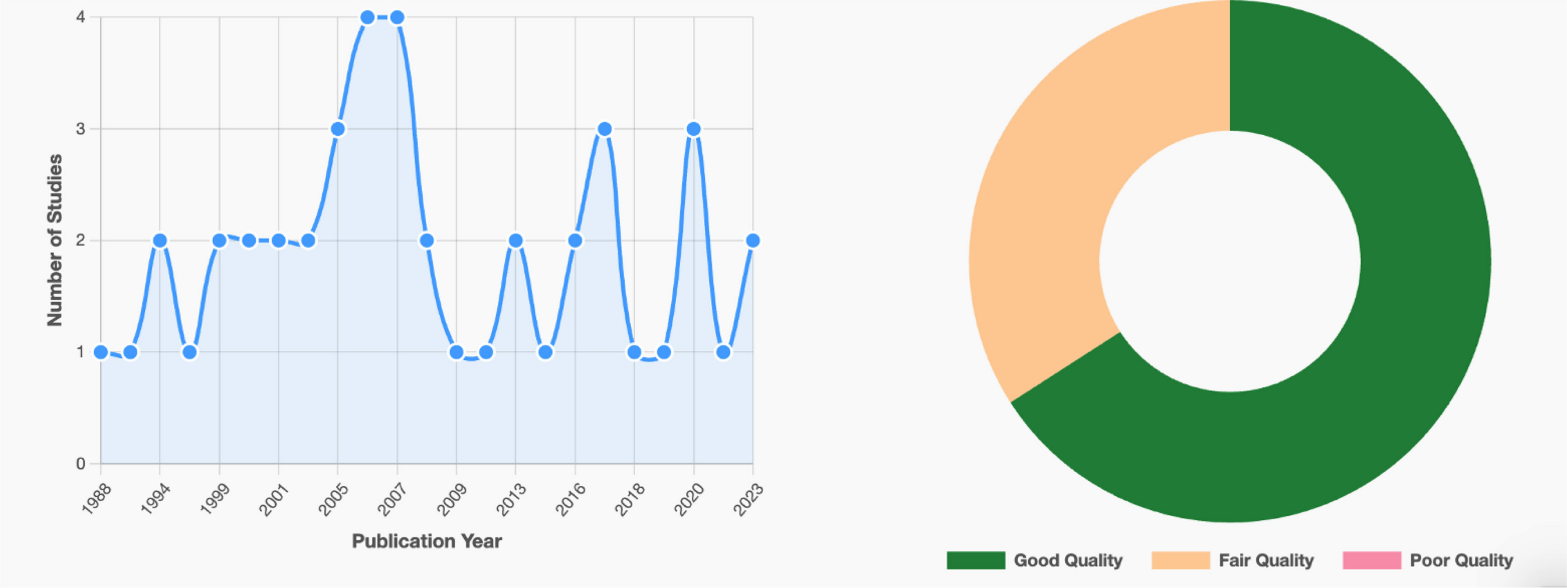
Publication year distribution and quality assessment results of studies.

The publication year of the included studies ranges from 1988 to 2023. Out of the analysed articles, 12 focused solely on risk factors for ADHD, while 23 investigated the risk factors for both ADHD and CD. Additionally, three articles specifically explored the risk factors for CD, and five articles examined the risk factors for IED alone. Only one article addressed the risk factors for both ADHD and IED together. Twenty‐nine studies were rated as good, 15 studies were rated as fair and no studies were rated as poor, suggesting that all included studies demonstrated a reasonable standard of quality for the purposes of this systematic review (see Figure 3). In the results section, we grouped the identified risk factors into the following categories. This classification is informed by the risk factor categorisation scheme, which provides a structured approach to grouping these factors (see Table 1).

**TABLE 1:** Risk factor categorisation.

Parental/caregiver–child relationships	Childhood violence exposure
	Poor quality early parenting
	Parenting style and parenting stress
	Maternal mental health problems
Obstetric and perinatal factors	Maternal smoking during pregnancy
	Maternal alcohol use during pregnancy
	Low birth weight
Sociodemographic factors	Adopted/foster care
	Low socioeconomic status family
Peer and school environment factors	Poor peer relationships
	Classroom misconduct
	Academic failure/learning difficulties
Aspect of the child	Children's biological susceptibility
	Children's specific personality traits

### 3.1 Parent/Caregiver–Child Relationships

#### 3.1.1 Childhood Violence Exposure

In this review, the family violence included witnessing domestic violence and being physically abused by a parent. In their pioneering research, Grizenko and Pawliuk (1994) presented compelling evidence that domestic violence substantially increases the likelihood of children developing ADHD, CD and impulse‐control disorders, quantified by an odds ratio (OR) of 16.8. It is important to recognise that the study was conducted at a time when the categorisation and understanding of impulse‐control disorders were less distinct than they are today. This lack of specificity could impact the study's precision in pinpointing the disorders in question, and its historical context should be considered when evaluating its relevance to current diagnostic standards and practices.

A later study conducted by Becker and McCloskey (2002) interviewed 287 mothers and found that exposure to domestic violence induced children to develop ADHD and CD. In this study, domestic violence was divided into marital violence and paternal abuse. Marital violence primarily affected children's attention problems, whereas paternal abuse primarily affected children's aggressive behaviour. The association indicated that experiencing physical abuse in childhood is a significant risk factor for the subsequent development of IED as well, as stated by Fanning et al. (2014). In a later German research study in which 156 young offenders were taken as the subjects, the researchers conducted a latent profile analysis and established a link between adverse childhood experiences (emotional abuse, emotional neglect, physical abuse, physical neglect and sexual abuse) and ADHD and IED in the forensic group (Barra et al. 2022). The results revealed that physical abuse predicted high ADHD symptomatology with co‐existing IED, but not without IED (B = 9.53, 95% CI: [5.35–13.70], *β* = 0.38, *p* < 0.001), which suggested a specific relationship between physical abuse and the co‐occurrence of ADHD and IED. However, the specific characteristics of the sample group should be noted as there is a distinct difference between the general sample and the forensic sample. A more recent study by Puhalla et al. (2020) investigated the impact of a history of childhood abuse and the presence of an alcohol use disorder on the development of IED. The findings suggested that while a history of childhood abuse was a unique predictor of IED, alcohol use disorder did not independently predict the condition.

In conclusion, multiple studies revealed that being physically abused/exposed to domestic violence is a risk factor for the later development of ADHD, CD and IED. However, the influence of gender on the association between domestic violence and the onset of these disorders remains a topic with mixed findings, necessitating further investigation to clarify these relationships.

#### 3.1.2 Poor Quality Early Parenting

Poor quality early parenting (PQEP), characterised by negative emotional exchanges, inconsistent behavioural supervision and arbitrary rule enforcement, was linked to moderate levels of concurrent symptoms of ADHD and CD, according to Lorber and Egeland's longitudinal study (2009). Although the association's statistical significance was marginal, PQEP's influence was enduring, persisting as a significant risk factor 26 years later. Notably, its predictive relevance was more pronounced during the developmental window of 2–5 years, suggesting a developmental stage‐specific impact, particularly during peaks of externalising behaviour. Furthermore, Shevidi et al. (2023) used the Parental Bonding Inventory (PBI) to assess PQEP and discovered that the PBI scores were lowest in the IED group compared with both the healthy control and psychiatric‐control groups, affirming PQEP as a risk factor for the later development of IED.

In synthesising these findings, it becomes apparent that PQEP stands as a shared risk factor for ADHD, CD and IED. Yet, a critical analysis indicates that the varied interpretations of PQEP may lead to a conflation of its components, obscuring the identification of the most impactful elements of early parenting. This recognition underscores the need for future research to dissect the components of PQEP with greater specificity to elucidate the nuances of its role in child development.

#### 3.1.3 Parenting Style and Parenting Stress

Two case–control studies of moderate size, DeWolfe et al. (2000) and DuPaul et al. (2001), both identified a correlation between parental stress and the occurrence of ADHD in children. However, the design of these studies does not permit conclusions about the direction of causality: whether parental stress contributes to the development of ADHD in children or if the challenges of managing ADHD symptoms in children lead to increased parental stress. In a later study, Meadows et al. (2007) analysed data from 2120 families collected through a national longitudinal survey to investigate the correlation between both maternal and paternal anxiety/depression and its potential links with the risk of ADHD in children. The findings indicated that maternal anxiety/depression was associated with an increased risk of ADHD. Conversely, paternal anxiety/depression did not show a significant association with the child's behavioural problems. In the study investigating the impact of maternal stress during the 1986 Chernobyl disaster on offspring behaviour disorders, researchers concluded that in utero exposure to the Chernobyl disaster, coupled with maternal anxiety presumably linked to that exposure, is associated with ADHD symptoms in 14‐year‐old adolescents. However, no association with CD was found (Huizink et al. 2007).

#### 3.1.4 Maternal Mental Health Problems

In a study by Lahey et al. (1989), the Minnesota Multiphasic Personality Inventory (MMPI) was administered to the biological mothers of 100 outpatients to examine any correlation between maternal personality characteristics and their children's diagnoses of ADHD and CD. The results showed no significant link between the mothers' personality disorders and their children's ADHD. However, CD was associated with maternal personality disorders, particularly with elevated scores on the hypochondriasis (Hs), paranoia (Pa) and mania (Ma) scales of the MMPI. Building on this, Grizenko and Pawliuk (1994) found that the presence of a depressive disorder in mothers was associated with ADHD and CD in their children (OR = 8.1). Dubois‐Comtois et al. (2013) partially supported these findings by demonstrating that higher maternal psychosocial distress significantly predicted ADHD and CD in children, with the mother–child relationship acting as a mediator. Additionally, Morgan et al. (2016) indicated that children raised by mothers with depressive symptoms had an increased risk for severe ADHD‐CD. Lastly, Meehan et al. (2017) suggested that maternal psychopathology, such as anxiety and depression, was partially responsible for the increased incidence of ADHD and CD in IC/ANX+ adolescents.

In conclusion, current research indicates that specific parenting styles and parenting stress are risk factors for both ADHD and CD. However, the literature lacks information on their influence on IED, presenting a significant opportunity for future research. Additionally, there is a scarcity of studies focusing on paternal influences compared with maternal, highlighting another area that requires further exploration.

### 3.2 Obstetric and Perinatal Factors

#### 3.2.1 Maternal Smoking During Pregnancy

Multiple studies have established a relationship between prenatal exposure to maternal smoking and a heightened occurrence of ADHD and CD in children. Initial research explored the link between prenatal smoking and various disorders. Through a retrospective case–control study, Hill et al. (2000) identified an association between prenatal smoking and CD and suggested that the previously reported association between maternal smoking during pregnancy and ADHD in offspring may not be supported when considering the influence of familial risk for alcoholism and prenatal alcohol use. The findings have not been uniformly consistent. Button et al. (2005) observed a connection between ADHD and maternal smoking, but this was not corroborated by Knopik et al. (2005), who found no association after adjusting for a comprehensive range of prenatal and parental risk factors. Conversely, Braun et al. (2006) conducted a robust large population–based cohort study and reported an association between prenatal smoking and ADHD. Further adding to the mixed results, two retrospective clinical cohort studies (Langley et al. 2007; Wakschlag et al. 2006) and one prospective cohort study (Weissman et al. 1999) presented somewhat conflicting evidence regarding the association between prenatal smoking and ADHD. Langley et al. suggested that maternal smoking in pregnancy and environmental adversity indexed by lower social class independently influence the clinical presentation of the ADHD phenotype.

While research has been conducted on the impact of maternal smoking on ADHD and CD, the results are not consistent. Inconsistencies may arise from varied methodologies, the nature of the data collection (whether retrospective or prospective) and the quantification of smoking habits. Additionally, the current body of literature does not address the potential effects of maternal smoking during pregnancy on IED, indicating a considerable gap for future investigation.

#### 3.2.2 Maternal Alcohol Use During Pregnancy

Similar to the effects of maternal smoking, maternal alcohol consumption during pregnancy has been established as a risk factor for adverse developmental outcomes in children.

Knopik et al. (2005) conducted a large‐scale twin study that demonstrated an increased rate of ADHD diagnoses in offspring of mothers who engaged in heavy alcohol consumption during pregnancy, even after accounting for various prenatal and parental risk factors. Supporting these findings are two additional studies. Bhatara et al. (2006), in a clinic‐based cohort study of children assessed for potential foetal alcohol spectrum disorders, observed a 41% prevalence of ADHD, which escalated with greater maternal prenatal alcohol exposure. Hill et al. (2000), through a smaller retrospective case–control study, reported a link between prenatal maternal alcohol use and CD; however, distinguishing this association from other possible confounding factors proved challenging for both studies.

In their study, Disney et al. (2008) investigated 1252 adolescents and their biological parents, finding that prenatal alcohol exposure correlated with an increased presentation of CD symptoms in the offspring. This association persisted even after adjusting for factors such as parental externalising disorders, monozygosity, gestational age and birth weight. Subsequent longitudinal research reinforced this finding, demonstrating a significant relationship between prenatal alcohol exposure and CD in adolescents, with the effect enduring even after accounting for environmental variables, maternal psychopathology and other prenatal exposures.

The accumulation of evidence suggests that maternal alcohol consumption during pregnancy is a significant risk factor for the development of ADHD and CD in children. Yet, there has been no exploration into the potential impact of maternal alcohol use on the emergence of IED, which presents a direction for future research.

#### 3.2.3 Low Birth Weight

The earliest study examined the association between low birth weight (LBW) and children's development of ADHD and CD identified in the search is Mick et al. (2002), in which they found that children with ADHD were three times more likely to have LBW than those without ADHD. Similarly, Sasaluxnanon and Kaewpornsawan (2005) discovered that the prevalence of ADHD in Thai children with a birth weight under 2500 g was 3.6 times higher than in the control group. Later, Stein et al. (2006) analysed data from the National Health Interview Survey, focusing on 7817 children with moderately low birth weight (MLBW, 1500–2499 g). However, the data were collected from parents or caregivers, and it might be subject to recall bias. The study revealed that MLBW children had a significantly higher likelihood of being diagnosed with ADD or ADHD compared with normal birthweight children (NBW), but not with CD.

A longitudinal study by Nigg and Breslau (2007) found that LBW predicted ADHD, even after controlling for several other familial characteristics. However, LBW was tested not to be associated with CD in this study as well. The authors concluded that while ADHD may occasionally serve as a precursor to CD, it maintained a partially separate trajectory with distinct underlying causes. The conclusion that LBW is a risk factor for ADHD has been reinforced by Scott et al. (2012), in which the researchers concluded that the rate of ADHD was about twice as high for the LBW group than for the NBW group (OR (95% CI) = 2.50 (1.34, 4.68), *p* = 0.004). Later, Ficks et al. (2013) also found a modest association between LBW and CD, ADHD. However, the researchers concluded that the overall magnitude of these associations was very minor, accounting for less than 1% of the variance in symptom dimensions. Therefore, they suggested that LBW is not a significant risk factor for ADHD or CD. However, a longitudinal study by Morgan et al. (2016) followed 7456 children from kindergarten to eighth grade and found that the percentage of children with LBW showing the development of ADHD and CD decreased from kindergarten to eighth grade.

These studies also examined the influence of children’s age and gender on the relationship between LBW and externalising disorders. Momany et al. (2017) indicated that LBW contributes to the development of ADHD and CD and that gender moderates this association, with stronger associations found in boys compared with girls. Collectively, these studies contribute to an understanding of LBW's connection to ADHD and CD. Although a modest link is sometimes present, its clinical relevance is disputed, with the potential variability attributed to sample sizes and gender's moderating effects—a point that continues to stir debate in the research community. However, the role of LBW, a well‐documented risk factor for ADHD and CD, has not been examined in the context of IED. This oversight suggests a potential area for future research, as establishing LBW as a risk factor for IED could significantly impact the early identification and intervention strategies for this disorder.

### 3.3 Sociodemographic Factors

#### 3.3.1 Adopted/Foster Care

Multiple studies discussed the influence of adopted/foster care experience on children's development of these three externalising disorders. The earliest study on adoption/foster care in this review can be dated back to a 1994 study, in which they utilised the Christchurch Psychiatric Epidemiological Survey (CPES) to examine the link between parental separation and the development of CD. The analysis of data from 935 children indicated that those who had gone through parental separation showed higher instances of CD. However, the apparent correlation between parental separation and these disorders diminished significantly once a range of social and contextual factors were controlled for in the analysis. A later 1995 retrospective study partially aligned with the previous conclusion. Sullivan et al. (1995) also utilised data from the CPES and highlighted a significant association between adoption and an increased incidence of CD in childhood.

In the 14‐year longitudinal study conducted by Moore and Fombonne (1999), they found that adopted children (both boys and girls) had a higher incidence of later ADHD and CD than children raised by their biological parents. For boys, a significant difference was found (*X* ^2^ = 98; *df* = 6; *p* = 0.003), which was largely explained by the overrepresentation of disruptive behaviour in the adopted group. For girls, a significant difference was also found for profiles of diagnosis (*X* ^2^ = 28.50; *df* = 6; *p* < 0.001) and reflected an increased frequency of ADHD and CD. A more recent study conducted by Humphreys et al. (2020) stated that children in adopted families or foster care systems are more likely to meet the diagnosis criteria for ADHD and CD. Shevidi et al. (2023) reached a conclusion that being separated from both biological parents will increase the child's chance of developing IED (OR = 0.54; compared with the HC + PC group).

In conclusion, separation from both biological parents (either in foster care or being adopted) is a shared risk factor for ADHD, CD and IED. However, the impact of being separated from biological parents and placed in alternative care settings can vary depending on various factors, such as the quality of care, stability of placement and individual resilience. Further research is needed to better understand the long‐term effects of adoption and foster care.

#### 3.3.2 Low Socioeconomic Status Family

In the review, financial indicators such as income and savings were combined with indicators of social status, such as parental education level, to assess socioeconomic standing. Multiple high‐quality articles indicated that coming from a LSES family is associated with children's development of ADHD, CD and IED. The earliest study included in this review dated to Lambert (1988). He stated that low SES status is a critical risk factor for children's conduct problems (0.148) and attention problems. Ficks et al. (2013) included the sample's family income as a covariate, and in the income X birth weight model, they found that children with the same LBW but who come from families with relatively low income have a higher chance of developing ADHD and CD, indicating that low family income is an environmental risk factor.

Morgan et al. (2016) stated that young children raised in low SES families and/or communities have higher risk for overall externalising disorders over time. In this study, children from lowest quintile SES families have the greatest risk of ADHD and CD. In the research of the personality traits of children with externalising disorders. Meehan et al. (2017) found that IC/ANX+ youth also have a higher chance of coming from socioeconomic disadvantaged backgrounds, indicating that LSES family could be an environmental risk factor for the later development of ADHD and CD. Recent study demonstrated that participants with IED have a lower mean socioeconomic score than both HC and PC (PC < HC) (Shevidi et al. 2023). However, this study only briefly noted LSES in the demographic data of participants without further investigating its role as a risk factor for IED.

In conclusion, LSES can be viewed as a shared risk factor for ADHD, CD and IED. However, LSES families are typically characterised as having an annual income below the national average or parents with low educational levels (Noble et al. 2015). While this definition provided a general framework for understanding LSES, it failed to capture the full complexity and multidimensional nature of socioeconomic status, which encompasses various factors beyond just income and education.

#### 3.3.3 Peer and School Environment

Lambert (1988) identified poor peer relationships and classroom misconduct as predictors for ADHD and CD in a study of 367 students, pinpointing interpersonal problems as the most frequent predictor for these conditions. However, the study's reliance on teacher assessments and student self‐reports, from a particular student cohort, may introduce biases and constrain the generalisability of the findings regarding peer interactions and behavioural issues. Further research by Grizenko and Pawliuk (1994) with a sample of 50 disordered and 50 controlled pre‐adolescents highlighted learning difficulties (*OR* = 26.3) and school failure (OR = 86.0) as significant risk factors for ADHD, CD and impulse control disorders.

Owens and Hinshaw (2016) found that academic failure and peer conflict, including school expulsions, were predictors of ADHD, CD and other externalising problems in young adulthood. However, the study did not precisely define ‘externalising problems,’ making it unclear if IED was included in the associated outcomes. The study's cross‐sectional design also precluded establishing causality between school‐related factors and subsequent behavioural disorders. Meehan et al. (2017) demonstrated that high‐anxiety IC children with worse performance in national standardised school tests and greater behaviour problems at school were at greater risk of developing ADHD and CD. Later, Shevidi et al. (2023) explored the predictive effects of learning difficulties, behavioural issues and peer relationships on the development of IED. Their findings indicated that participants with IED had a higher reported history of learning difficulties in school compared with healthy controls and a history of fighting with peers before the age of 10 was notably higher among IED participants (adjusted OR: 3.09), pointing towards a significant association.

In summary, a variety of peer‐ and school‐related risk factors—including peer rejection, classroom misconduct, poor academic performance and conflicts with peers—have been recognised as shared risk factors for ADHD, CD and IED.

### 3.4 Aspect of the Child

#### 3.4.1 Children's Biological Susceptibility

Multiple studies explored the biological underpinnings of ADHD, CD and IED, seeking to unravel potential genetic predispositions for these disorders. The 2008 international multicentre genetic study scrutinised over a million autosomal markers for links to conduct problems yet failed to achieve genome‐wide statistical significance (*p* < 5 × 10^−7^), leaving candidate genes for ADHD and CD unidentified (Anney et al. 2008). Subsequently, research by Ficks et al. (2013) linked variations in the monoamine oxidase A (MAOA) and catechol‐O‐methyltransferase (COMT) genes to impulsivity and inattention in children, suggesting a genetic foundation for the later development of ADHD and CD. The most recent advances in genome‐wide association studies (GWAS) have shed light on IED, identifying 27 CpG sites with differential methylation patterns in those with the disorder. These sites correspond to genes integral to inflammation and immunity, endocrine function and neuronal differentiation, implicating the inflammatory response as a key risk factor for IED (Montalvo‐Ortiz et al. 2018). Inflammation has also been identified as a significant risk factor for ADHD, as highlighted in Dunn et al. (2019). This research underscored a notable association between ADHD and peripheral inflammation, a link further corroborated by animal models demonstrating a connection between developmental exposure to inflammation and ADHD. Lastly, a 2023 study proved that the inflammatory response is a risk factor for both ADHD (OR = 1.27, 95% CI: [1.07, 1.49]) and CD (OR = 1.50, 95% CI:[1.24, 1.81]) (Gibson et al. 2023).

In conclusion, although no shared risk genes have been identified for the development of these three disorders, we successfully realised that the three disorders all emerged from biological mechanisms, such as inflammation. Future research should focus on identifying the full spectrum of biological pathways for these disorders.

#### 3.4.2 Children's Specific Personality Traits

Multiple high‐quality studies investigated the role of children's specific personality traits as risk factors for ADHD, CD and IED. Research indicated that the personal characteristics of the child are an important class of vulnerability factors in the face of adversity. Meehan et al. (2017) suggested that children with interpersonal callousness (IC)/high anxiety traits (ANX+) were more likely to develop ADHD and CD (all *p* < 0.001). Another research study conducted by Mann et al. (2020) concluded that callous‐unemotional (CU) traits, which are defined as low empathy, IC, constrained effect and a lack of performance‐related concern, were strongly associated with children's development of ADHD, CD and IED. Fanti et al. (2017) reached a similar conclusion, stating that children with a stable high CU trajectory were more likely to manifest high and stable levels of CD and ADHD, and this study suggested that the association between CU trajectory and these symptoms strengthened across the lifespan; a more robust conclusion was presented than in previous studies.

In conclusion, children with certain personality traits are more vulnerable to the development of ADHD, CD and IED. While the studies discussed provide valuable insights into the potential relationship between specific personality traits and the development of externalising disorders, it is important to acknowledge that the body of research in this area remains limited.

### 3.5 Shared Influence Among the Disorders

Based on the literature review, we identified several risk factors that significantly contribute to the development of ADHD, CD and IED. Childhood maltreatment is a key factor, with physical abuse and family violence being particularly impactful in the development of these disorders. Moreover, both poor‐quality early parenting and low socioeconomic status families are common risk factors for all three disorders. Studies have shown that growing up in unstable, resource‐poor families can negatively affect children's development. This is also true for children who are adopted or placed in foster care, where the developmental impact is partly due to environmental influences. In addition to family dynamics, school‐related risk factors also play a significant role. Poor relationships with classmates and academic failure both increase the likelihood of developing these disorders. This exploration of shared risk factors underscores the critical role of family environment in these disorders and suggests that future interventions should focus on family‐based approaches. Another important shared risk factor is the inflammation response in children. Children born with placental inflammation are at a heightened risk of developing ADHD, CD and IED. This finding has significant implications for future research and underscores the importance of early detection, monitoring and intervention in these children.

### 3.6 Unique Influence Among the Disorders

**FIGURE 4:**
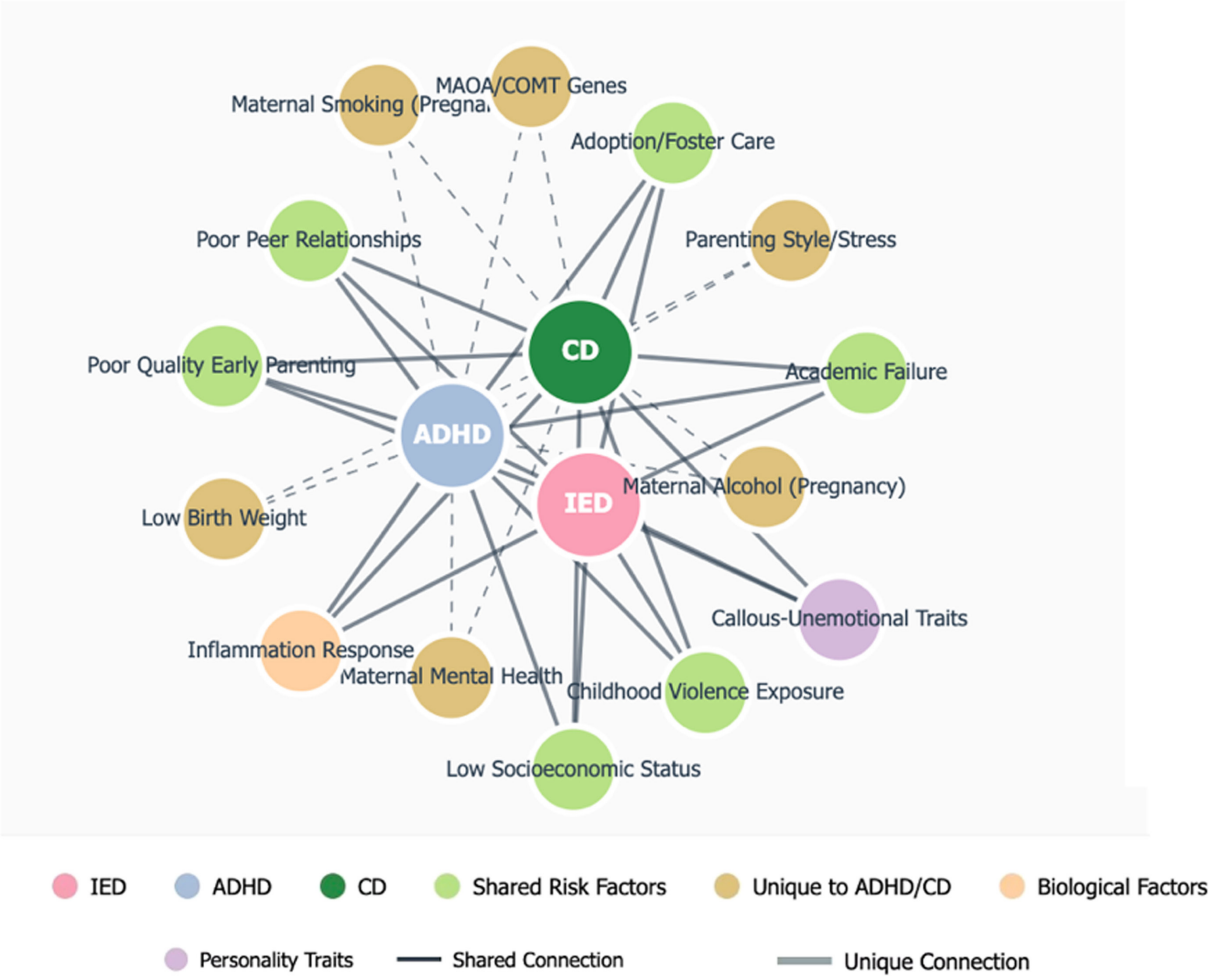
Network and Venn diagram of risk factors for ADHD, IED and CD. The network diagram depicts the interconnectedness of risk factors, highlighting shared influences between disorders (represented by thick edges) and unique connections (represented by thin edges).

For ADHD, CD and IED, we attempted to define unique risk factors as those that have been evaluated across these disorders but are only associated with one (see in Table 2). However, it was challenging to pinpoint such factors since most contributed to at least two of the disorders. Nonetheless, we have identified several key unexplored risk factors for each disorder, which offer directions for future research. Parenting stress, examined as a risk factor for both ADHD and CD, leaves a notable gap in the literature regarding its association with IED. Considering IED is characterised by intense anger outbursts and stress, it is plausible to hypothesise its connection to being raised in a stressful environment. Future studies should investigate this potential relationship. Another unexplored area for IED is obstetric and perinatal factors. Various risks in this category, such as maternal smoking and drinking during pregnancy and LBW, have been linked to ADHD and CD. Yet, their impact on IED remains unexplored. Given the high comorbidity rate, investigating environmental influences alongside pre‐birth and biological risk factors could be beneficial (see in Figure 4).

**TABLE 2:** Summary of developmental risk factors for ADHD, conduct disorder and IED across key domains.

Factor/domain	ADHD	CD	IED	Notes (design/typical sample)
Childhood violence/maltreatment	Strong	Strong	Limited	ADHD/CD: multiple longitudinal, adjusted; IED: few cross‐sectional, forensic/clinical
Low socioeconomic status/poverty	Strong	Strong	Limited	IED mostly unadjusted or small samples
Harsh/low‐warmth parenting	Strong	Strong	Limited	IED parenting rarely measured explicitly
Parental psychopathology (maternal/paternal)	Strong	Strong	NS	No IED study with clear parental diagnoses
Prenatal/perinatal complications	Strong	Limited	NS	IED: not studied
Peer delinquency/deviant peers	Limited	Strong	NS	IED: not studied
Adoption/alternative care	Limited	Limited	NS/inconsistent	Mixed directions, small n
School‐related disadvantage	Strong	Limited	NS	IED: no data
Biological/inflammatory markers	Limited	Limited	NS	Reported sporadically, no cross‐disorder comparison

## 4 Discussion

To move beyond parallel descriptions of ADHD, CD and IED, we synthesised the identified risk and contextual factors according to the consistency and depth of evidence across the three disorders. Three patterns emerged. First, a small cluster of adversities showed convergent and relatively strong evidence across all three conditions, particularly exposure to childhood violence or maltreatment, low socioeconomic status and suboptimal parenting (low warmth, high harshness). For ADHD and CD, these factors were supported by multiple longitudinal or prospective cohort studies with statistical adjustment for key confounders; for IED, evidence came from fewer, often cross‐sectional clinical or justice‐involved samples, but the direction of association was comparable. We therefore considered these factors to have strong cross‐disorder support. Second, several factors demonstrated substantial evidence for ADHD and/or CD but remained unexamined or very sparsely examined in IED. These included prenatal/perinatal complications, parental psychopathology (especially paternal), peer delinquency and school‐related disadvantage. In ADHD and CD, these correlates have been replicated across designs and populations, whereas for IED, we found no study or only single reports without adequate control. We therefore classified these as having limited cross‐disorder evidence, indicating that an extension of existing ADHD/CD designs to include IED measures would be highly informative. Third, a small number of variables showed inconsistent or disorder‐specific patterns (e.g., adoption status, certain temperament dimensions), where effect directions diverged across studies or were present for one disorder but absent for the others. This graded appraisal highlights that the apparent “shared risk architecture” for externalising conditions is best supported for distal adversities and parenting‐related factors, while biologically or developmentally more specific mechanisms have largely not been evaluated for IED.

### 4.1 Integrated Developmental–Ecological Interpretation

Rather than treating the correlated risk factors model, dimensional adversity frameworks and biological embedding as separate explanations, our findings can be organised into a three‐level pathway. Level 1 (distal adversity) comprises broad, well‐replicated contextual risks: childhood violence and maltreatment, low socioeconomic status, suboptimal or harsh parenting and, in some studies, adoption or alternative care. These exposures were documented most consistently for ADHD and CD, and although more sparsely, were also observable among individuals with IED, indicating that all three externalising conditions are anchored in a partly shared adversity context.

Level 2 (partially shared biological and developmental embedding) captures mechanisms through which these early adversities may become instantiated via prenatal/perinatal insults, stress–inflammation pathways or candidate dopaminergic/serotonergic systems (e.g., MAOA/COMT), which have been explored repeatedly in ADHD and, to a lesser extent, CD. For IED, however, this level is strikingly underdeveloped: Very few studies examined biological or developmental mediators that would link early adversity to later explosive aggression. This uneven coverage means that apparent “differences” between IED and the other two disorders may, at present, simply reflect a missing middle layer of evidence rather than genuinely distinct aetiologies.

Level 3 (disorder‐specific expression) refers to how these broadly similar upstream liabilities are channelled into different clinical phenotypes: attentional and executive deficits and school impairment in ADHD; rule‐breaking, deviant peer affiliation and conduct problems in CD; and recurrent anger outbursts and low threshold for aggression in IED. On this view, what looks like disorder‐specific risk may in fact be a disorder‐specific *realisation* of a largely overlapping adversity–biology platform.

**FIGURE 5:**
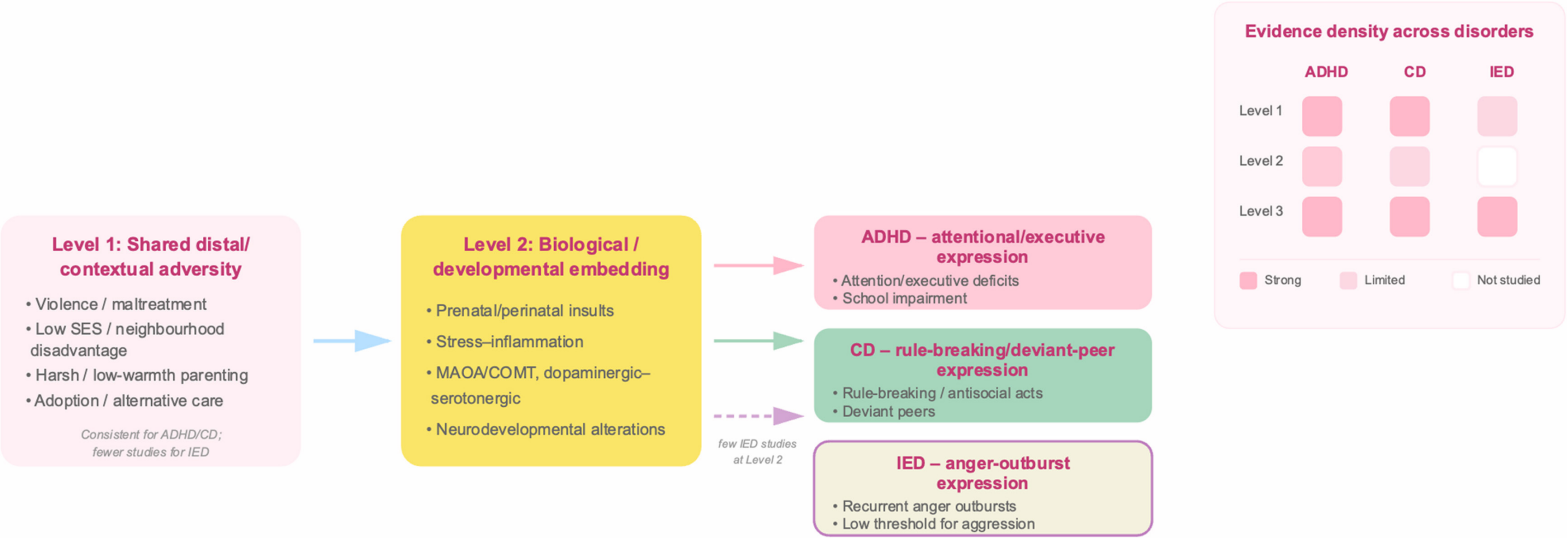
Three‐level adversity–embedding–expression framework across ADHD, CD and IED. This figure illustrates a proposed three‐level pathway whereby shared distal/contextual adversity (Level 1) is biologically/developmentally embedded (Level 2) and subsequently expressed as disorder‐specific phenotypes (Level 3) in ADHD, CD and IED. The evidence‐density inset summarises the relative strength of published evidence at each level by disorder (dark pink = strong, lighter pink = limited, white = not studied).

Framing the literature in this tiered way clarifies our main conclusion: The available evidence most robustly supports shared distal adversity (Level 1) and, for ADHD/CD, some degree of shared embedding (Level 2), but IED is currently substantiated almost exclusively at Level 1. Consequently, the difficulty of identifying ‘unique influences’ for IED is not necessarily because they do not exist; it is because the evidence base has not yet filled in Level 2 for this disorder. Future studies that extend biological, prenatal or longitudinal designs routinely used in ADHD/CD to samples with IED will therefore be crucial for testing whether IED follows the same adversity → embedding → expression sequence or whether it diverges at the mechanistic stage (see Figure 5).

However, there are notable inconsistencies in the findings that warrant further exploration. For instance, the relationship between maternal smoking and the development of ADHD and CD remains inconclusive. Initial studies, such as Hill et al. (2000), identified a link between prenatal smoking and CD, but subsequent research has yielded mixed results. Variations in study methodologies, sample populations and measurement approaches may explain these discrepancies. Additionally, the role of LBW as a risk factor for ADHD and CD is similarly debated. Some studies report a modest association between LBW and externalising behaviours, while others question its clinical relevance. Notably, the influence of LBW on the development of IED remains unexplored, which presents a critical gap for future research.

The inflammatory response, a key mechanism linking childhood adversity to later mental health problems, was consistently identified as a significant risk factor across all three disorders. Previous research has linked the inflammatory response to the development of psychological disorders in late childhood, yet the specific mechanisms of this association are not fully understood. A meta‐analysis examining the relationship between childhood psychological stress and inflammatory response concluded that stress in childhood can be a risk factor for chronic illnesses in adulthood. Notably, inflammation has been identified as a potential mechanism that underlies this vulnerability to chronic illness. The influence of early‐life psychological stressors on various mental health conditions across an individual's lifetime has been extensively studied (Schneiderman et al. 2005; Toussaint et al. 2016). These studies suggest that inflammatory responses may act as a physiological reaction to childhood stressors. According to the Biological Embedding of Childhood Stress Model, childhood stressors can induce a pro‐inflammatory phenotype in two ways (Ehrlich et al. 2016): firstly, through programming effects, where adversity becomes permanently ingrained in cellular functions during crucial periods of immune development; secondly, through accentuating effects, where adversity influences trajectories of psychosocial and behavioural development in ways that sustain inflammation throughout life.

### 4.2 Practical Implications and Future Directions

From a practical standpoint, this deeper understanding is essential for the development of targeted interventions that can address the specific needs of individuals with these disorders. Currently, intervention strategies for ADHD and CD often involve multi‐contextual approaches that include family therapy, parenting training and school‐based interventions. Studies have shown that these approaches can be highly effective in mitigating the symptoms and improving outcomes for children with ADHD and CD (Feng et al. 2023). Family therapy, in particular, is instrumental in addressing the familial dynamics that contribute to these disorders, while parenting training helps caregivers develop skills to manage behavioural problems. In light of the largely familial and contextual risk architecture we identified, we recommend adapting existing parent‐management/parent‐training protocols used in ADHD/CD to an IED‐specific ‘emotional‐outburst–contextualised’ module. This module should (i) help caregivers recognise child‐specific anger/trigger patterns and early physiological or situational cues, (ii) teach de‐escalation and emotion‐coaching techniques to be used in the moment of an outburst and (iii) establish consistent, pro‐social family communication and problem‐solving routines that can be coordinated with school or justice‐involved settings when relevant. Because these components are already well‐supported in externalising‐disorder interventions, they are realistically transferable to IED populations.

Moreover, one significant challenge in the field is the ongoing revisions and debates surrounding the diagnostic criteria for IED. As highlighted by Coccaro et al. (2014) and other experts, the boundaries of IED in relation to other externalising disorders, such as CD and oppositional defiant disorder (ODD), remain subject to change. This evolving nature of IED's diagnostic criteria underscores the importance of broadening the symptomatology spectrum in research, as our study has done, to reduce potential biases and provide a more comprehensive understanding of the disorder. A more inclusive diagnostic framework will allow clinicians to better differentiate IED from other disorders that share similar symptoms, thus improving the accuracy of diagnosis and treatment planning.

Our systematic review also identifies specific shortcomings in IED research. Despite recent increased attention to IED, significant gaps remain. By analysing the impact of parental personality traits and mental health on ADHD and CD, we have observed a profound influence of these factors on children's externalising disorders. It is imperative for future studies to explore whether similar patterns are evident in IED. We also recognise a notable limitation in the paternal aspect of IED research, paralleling trends in ADHD and CD studies. The predominant focus on maternal influences, potentially stemming from traditional views of parental roles (highlighted in works by Davé et al. 2008; Leahy‐Warren et al. 2023; Phares 1992), overlooks the equally significant role fathers play in child development. Future research should therefore adopt a more balanced perspective, incorporating insights from both parents.

Furthermore, the gender‐specific and developmental trajectories of these risk factors need to be considered in future research. Studies examining the moderating role of gender in the relationship between environmental stressors and disorder development have produced mixed results. Some research suggests that boys may be more susceptible to the effects of early adversities, such as exposure to family violence, while others report no gender differences. Understanding how gender influences these relationships could help in designing more targeted interventions. Similarly, the role of developmental timing in the impact of these risk factors needs further exploration. Research suggests that early‐life exposure to adversity may have different effects depending on the developmental stage, with certain windows of vulnerability potentially exerting more profound long‐term effects.

### 4.3 Limitations

This study, despite being the first systematic review attempting to synthesise the literature on risk factors for IED, ADHD and CD, has certain limitations that require critical consideration. First, the studies included in this review are varied in design, ranging from longitudinal studies to cross‐sectional studies. While this diversity enriches the breadth of the findings, it also introduces inherent challenges when comparing results across studies. Cross‐sectional designs, in particular, limit the ability to draw causal inferences between risk factors and disorder development. Second, although gender was briefly discussed, it was not systematically explored in terms of how risk factors for ADHD, IED and CD might differ between boys and girls. Research indicates that boys and girls may experience and respond to environmental stressors differently, with gender potentially influencing the severity and manifestation of symptoms. Given the distinct symptomatology observed in each disorder, it is important to examine how gender mediates the impact of shared risk factors on disorder development. Third, although the biological embedding model offers a useful way to connect distal adversity with later behavioural dysregulation, the empirical support for a *causal* chain ‘childhood stress → persistent inflammation → externalising disorder’ is still provisional. Most of the studies we cite measure adversity in retrospect and inflammatory markers at a single timepoint, which precludes formal mediation testing and makes residual confounding by socioeconomic context, health behaviours, or ongoing stress likely. Moreover, the relation may be bidirectional: dysregulated, aggressive or impulsive behaviour can elicit recurrent interpersonal stress and health‐risk behaviours that themselves sustain low‐grade inflammation over time. A stronger test will require prospective cohorts that (i) assess adversity in childhood, (ii) obtain repeated inflammatory and epigenetic indices during sensitive periods of immune development and (iii) follow participants into the developmental windows in which ADHD, CD and especially IED tend to manifest, allowing formal longitudinal mediation. Finally, inflammation is unlikely to act in isolation; integrating HPA‐axis activity, autonomic reactivity and fronto‐limbic circuit measures would allow a more ecologically valid account of how adversity becomes ‘embedded’.

## 5 Conclusion

The review provides valuable insights and highlights important implications for future research, policy and practice. It points to the need for more inclusive research, particularly involving diverse populations beyond Western and developed countries, to better understand risk factors for disorders like IED. Moreover, the review calls for examining potential moderators and mediators to elucidate the intricate connections between risk factors and externalising disorders. This approach could improve the effectiveness of prevention programs by moving away from broad, nonspecific strategies and focusing on more targeted interventions considering these moderating and mediating influences. By synthesising research on shared risk factors, it was observed that these relationships vary depending on the child's gender and developmental stage. Future research could benefit from a life course perspective, using prospective designs with repeated assessments to deepen our understanding of how childhood risk factors evolve into chronic externalising disorders. For at‐risk groups, family‐based interventions might be particularly beneficial. The current findings offer guidance on the timing and setting of such interventions, although further research is needed to confirm and expand these insights.

## Supporting information


**Appendix A:** Supporting Information.

**Appendix B:** Supporting Information.

**Appendix C:** Supporting Information.

**Appendix D:** Supporting Information.

## Data Availability

The data that support the findings of this study are available on request from the corresponding author. The data are not publicly available due to privacy or ethical restrictions.
