# Supplementary material for: Genetic and Environmental Risk Factors for Intermittent Explosive Disorder, ADHD and Conduct Disorder: Shared and Unique Influences
Source: Clin Psychol Psychother. 2025 Dec 15;32(6):e70195. doi: 10.1002/cpp.70195 (PMC12706422; doi:10.1002/cpp.70195)
Supplement: Supplementary file 2 — Appendix B: Supporting Information. [file CPP-32-e70195-s004.docx]

**Appendix B. Full Electronic Search Strategy**

This appendix describes the complete search strategy used for the systematic review on risk and antecedent factors associated with intermittent explosive disorder (IED), attention-deficit/hyperactivity disorder (ADHD), and conduct disorder (CD). Searches were run in August 2025 and the syntax was adapted to the requirements of each database. No country restrictions were applied. Human studies from 1988 onwards were considered.

## Table 1 Databases and Platforms Searched (August 2025)

| Database / Platform | Coverage / Notes |
| --- | --- |
| MEDLINE (Ovid) | All years to August 2025 |
| Embase (Ovid) | All years to August 2025 |
| APA PsycINFO | All years to August 2025 |
| PubMed | All years to August 2025 |
| Web of Science Core Collection | SCI-EXPANDED, SSCI, A&HCI, ESCI; 1988–2025 |
| Cochrane Library (incl. CENTRAL) | Trials and reports, to August 2025 |

Note. Databases were selected to cover biomedical, psychological, and interdisciplinary literature consistent with APA guidelines on reporting information sources.

## Table 2 Ovid MEDLINE (Ovid) Search Strategy

| Line | Search term |
| --- | --- |
| 1 | exp Attention Deficit Disorder with Hyperactivity/ |
| 2 | (ADHD or "attention deficit hyperactivity disorder*" or "attention-deficit/hyperactivity disorder*" or "hyperkinetic disorder*").ti,ab,kw. |
| 3 | 1 or 2 |
| 4 | exp Conduct Disorder/ |
| 5 | ("conduct disorder*" or (CD adj2 (child* or adolescen* or youth))).ti,ab,kw. |
| 6 | 4 or 5 |
| 7 | Intermittent Explosive Disorder/ |
| 8 | ("intermittent explosive disorder*" or IED or "impulsive aggression" or "impulsive aggressive" or "impulse control disorder*" or "explosive anger" or "anger outburst*" or "violent outburst*").ti,ab,kw. |
| 9 | 7 or 8 |
| 10 | 3 or 6 or 9 |
| 11 | exp Risk Factors/ |
| 12 | exp Etiology/ |
| 13 | exp Child Abuse/ or exp Domestic Violence/ or exp Adverse Childhood Experiences/ |
| 14 | exp Socioeconomic Factors/ |
| 15 | exp Parenting/ or Parent-Child Relations/ |
| 16 | exp Pregnancy Complications/ or exp Prenatal Exposure Delayed Effects/ or exp Fetal Alcohol Spectrum Disorders/ |
| 17 | exp Infant, Low Birth Weight/ |
| 18 | exp Inflammation/ or exp Cytokines/ |
| 19 | 11 or 12 or 13 or 14 or 15 or 16 or 17 or 18 |
| 20 | ("risk factor*" or "risk profile*" or predictor* or antecedent* or determinant* or correlat* or "vulnerability factor*" or "environment* exposure" or "early-life adversity" or "childhood maltreat*" or "physical abuse" or "domestic violence" or "family violence" or "poor parenting" or "harsh parenting" or "low-warmth parenting" or "parenting stress" or "maternal mental health" or "maternal depression" or "prenatal smoking" or "maternal smoking" or "prenatal alcohol" or "maternal alcohol" or "fetal alcohol" or "low birth weight" or "socioeconomic status" or "low SES" or poverty or "adopt* care" or "foster care" or "peer problem*" or "school failure" or "academic failure" or "classroom misconduct" or "learning difficult*" or inflammation or "pro-inflammatory" or "MAOA" or "COMT").ti,ab,kw. |
| 21 | 19 or 20 |
| 22 | exp Humans/ |
| 23 | 10 and 21 |
| 24 | 23 and 22 |
| 25 | limit 24 to (journal article and yr="1988 -Current") |

Note. Field tags (ti,ab,kw.) were used to search titles, abstracts, and keywords. Subject headings were exploded where available. The final line restricted results to human journal articles from 1988 to the search date.

## Table 3 PubMed (NIH) Search Strategy

| Search string |
| --- |
| (("Intermittent Explosive Disorder"[Mesh] OR "intermittent explosive disorder"[tiab] OR IED[tiab] OR "impulsive aggression"[tiab] OR "impulse control disorders"[Mesh] OR "explosive anger"[tiab]) OR ("Attention Deficit Disorder with Hyperactivity"[Mesh] OR ADHD[tiab] OR "attention deficit hyperactivity disorder"[tiab]) OR ("Conduct Disorder"[Mesh] OR "conduct disorder"[tiab] OR "conduct problems"[tiab])) AND (("Risk Factors"[Mesh] OR "Etiology"[Subheading] OR "Child Abuse"[Mesh] OR "Domestic Violence"[Mesh] OR "Socioeconomic Factors"[Mesh] OR "Parenting"[Mesh] OR "Pregnancy Complications"[Mesh] OR "Prenatal Exposure Delayed Effects"[Mesh] OR "Infant, Low Birth Weight"[Mesh] OR "Inflammation"[Mesh]) OR ("risk factor*"[tiab] OR predictor*[tiab] OR antecedent*[tiab] OR determinant*[tiab] OR "childhood maltreat*"[tiab] OR "family violence"[tiab] OR "low socioeconomic status"[tiab] OR poverty[tiab] OR "harsh parenting"[tiab] OR "parenting stress"[tiab] OR "prenatal smoking"[tiab] OR "prenatal alcohol"[tiab] OR "fetal alcohol"[tiab] OR "low birth weight"[tiab] OR "foster care"[tiab] OR adoption[tiab] OR "peer problem*"[tiab] OR "school failure"[tiab] OR inflammation[tiab] OR "pro-inflammatory"[tiab])) Filters: Humans; 1988/01/01–2025/08/31. |

Note. The PubMed search was adapted from the Ovid MEDLINE strategy by mapping Medical Subject Headings (MeSH) to their PubMed equivalents and by using [tiab] for free-text terms. Filters were used only at the end of the search.
