# Supplementary material for: Genetic and Environmental Risk Factors for Intermittent Explosive Disorder, ADHD and Conduct Disorder: Shared and Unique Influences
Source: Clin Psychol Psychother. 2025 Dec 15;32(6):e70195. doi: 10.1002/cpp.70195 (PMC12706422; doi:10.1002/cpp.70195)
Supplement: Supplementary file 3 — Appendix C: Supporting Information. [file CPP-32-e70195-s002.docx]

| **Study** | **Study design** | **Participants/methods** | **Risk factors** | **Disorders** | **Objectives** | **Results** |
| --- | --- | --- | --- | --- | --- | --- |
| 1. **Anney et al. 2008** | Cross-sectional | 938 individuals with ADHD and 208 individuals with CD | Genetic | ADHD, CD | Identify candidate genes that are important in ADHD and conduct disorders | No genome-wide statistical significance (P < 5 x 10(7)) was found for any of the tested markers and the three conduct problem traits |
| 1. **Barra et al. 2022** | Cross-sectional | 156 participants (129 males, 82.7%; 27 females, 17.3%) between 14 and 25 years (M=18.53 years, SD=2.13 years) | Adverse childhood experience | ADHD, IED | Gain sophisticated insights into the occurrence and associations of ADHD, IED, ACEs, and further psychiatric/ psychological impairments in young (male and female) offenders | ACEs predicted high ADHD symptomatology with co-existing IED, but not without IED. Because ACEs were positively associated with the occurrence of ADHD/IED and ADHD is one important risk factor for on-going criminal behaviours |
| 1. **Becker& McCloskey (2002)** | Longitudinal | During 1990–1991, 363 mother–child pairs were interviewed to assess the impact of marital violence on children’s mental health. These families were followed up during 1996–1997, and 299 youth were reinterviewed (an 83% retention rate) | Childhood exposure to violence | ADHD,  CD | Examine the impact of family violence on the development of attention and conduct problems in girls and boys | Family violence related to attention and conduct problems in girls only. Girls who displayed these problems in childhood were not necessarily at risk for later delinquency. Family violence in childhood had a direct effect on delinquency in girls. Boys who developed attention problems were more likely to show conduct problems that eventually led to adolescent delinquency. Family violence failed to account for problems or delinquency in boys. |
| 1. **Bhatara et al. (2006)** | Case control | The charts of 2231 youths referred for fetal alcohol spectrum disorders were reviewed. 1258 males and 957 females | Maternal alcohol use during pregnancy | ADHD | Examine the association between prevalence of ADHD and levels of risk for gestational alcohol exposure. | Overall prevalence of ADHD was 41%; Prevalence rates increased in accordance with the higher risk of gestational alcohol exposure. |
| 1. **Braun et al. (2006)** | Longitudinal | Data were obtained from the national health and nutrition examination survey 1999–2002. 4,704 children 4-15 years of age | Maternal smoking during pregnancy | ADHD | Examine the association of exposures to tobacco smoke and environmental lead with ADHD. | Prenatal tobacco exposure was significantly associated with ADHD.  Post-natal tobacco smoke exposure was not associated with ADHD. |
| 1. **Button et al. (2005)** | Population-based twin register | The study involved participants from CaStaNET (Cardiff Study of All Wales and Northwest England Twins), which is a comprehensive twin register. The twins selected for this research were school-aged, born between 1980 and 1991and these twins were between 5 and 18 years old. Out of 2,846 families who received questionnaires, a significant 73% (2,082 families) returned them completed. | Maternal smoking during pregnancy | ADHD | Examine the relationship between maternal smoking during pregnancy, antisocial behaviour, and ADHD in offspring. | Antisocial behaviour and ADHD symptoms were independently influenced by maternal prenatal smoking during pregnancy. |
| 1. **DeWolfe et al. (2000)** | Case-control | Twenty-five children (21 males, 4 females; mean age 4.8 years) with attention-deficit-hyperactivity disorder (ADHD) participated in this study in addition to 25 typically developing children (21 males, 4 females; mean age 4.9 years) | Parental stress | ADHD | Examine and compare the behavioral, social, and familial characteristics of preschool children with attention-deficit–hyperactivity disorder (ADHD) against those of typically developing peers. | Children in ADHD group rated as being more aggressive, demanding and less socially skilled.  Parents of the ADHD children rated themselves as less competent and had a more stressful restricted parenting role. |
| 1. **Disney et al. (2008)** | Population-based twin ascertainment method | A population sample of 1252 adolescents (53.8% female; drawn from the Minnesota Twin Family Study) as well as both their parents | Maternal alcohol use during pregnancy | CD | Examine the relationship between alcohol exposure in pregnancy and offspring conduct disorder (CD) symptoms in adolescence | Prenatal exposure to alcohol was associated with higher levels of CD symptoms in offspring, even after statistically controlling for the effects of parental externalizing disorders (i.e., illicit substance use disorders, alcohol dependence, and antisocial/behavioral disorders), prenatal nicotine exposure, monozygosity, gestational age, and birth weight. |
| **9.Dubois-Comtois et al. (2013)** | Longitudinal | Participants were 243 French-speaking mother-child dyads (122 girls) who were part of an ongoing longitudinal project | Parenting Stress | ADHD, CD | Examine the longitudinal relation between early school-age measures of maternal psychosocial distress, quality of mother-child interactions, and child attachment and behavior problem profiles in middle childhood using a multi-informant design. | Higher maternal psychosocial distress and controlling attachment patterns, either of the punitive or caregiving type, significantly predicted membership in both child internalizing and externalizing clinical problem groups. Lower mother-child interactive quality, male gender, and child ambivalent attachment were also predictors of ADHD and CD. |
| **10.Dunn et al., (2019)** | Longitudinal | Data came from the Accessible Resource for Integrated Epigenomic Studies, a subsample of mother-child pairs from the Avon Longitudinal Study of Parents and Children (n = 691-774). | Genetic | ADHD | The primary aim of this research is to investigate the sensitivity of DNA | There is a notable association between ADHD and peripheral inflammation, a link further corroborated by animal models demonstrating a connection between developmental exposure to inflammation and ADHD |
| **11.DuPaul et al. (2001)** | Case control | 94 children (58 with ADHD, 36 normal controls) between 3 and 5 years old participated | Parenting Stress | ADHD | To examine differences in home, school, and medical functioning between preschool-age children with ADHD and normal control children. | Children in the ADHD group had more problem behaviour and less social skills. Parents of the ADHD children experienced more stress and coped less adaptively and displayed more negative behaviour towards their children. |
| **12.Fanning et al. (2014)** | Case Control | 648 medically healthy subjects participated in this study as research volunteers. Ranged in age from 18 to 70 (M = 34.0, SD = 9.8) | Childhood maltreatment | IED | Examine the role of childhood maltreatment in children’s development. | Physical abuse in childhood was independently associated with IED |
| **13. Fanti et al. (2017)** | Longitudinal | Data were collected from a large sample of families in Cyprus at three time points, 1 year apart. Initially data was collected from 1,311 mothers and fathers (children’s mean age at study commencement = 9.38, SD = 1.04; 53.4% female) at Time 1 | Callous- unemotional traits | ADHD, CD | Examine developmental heterogeneity in callous-unemotional (CU) traits in a large sample of school-age children in Cyprus. | Repeated analysis of variance suggested that increases and decreases in CU traits were associated with similar changes in ADHD and CD. |
| 1. **Fergusson et al. (1994)** | Longitudinal cohort study | The data were gathered from the Christchurch Health and Developmental Study, which is a longitudinal study of a birth cohort of 1265 New Zealand children who have been studied at birth, 4 months, 1 year and annual intervals to the age of 15 years | Low SES family | ADHD, CD | Examine the life history of a small group of adolescents (3%) who were identified during a longitudinal study of a birth cohort of New Zealand children as displaying multiple problem behaviours at the age of 15 years. | The statistical profiles of these young individuals with conduct disorder revealed that many originated from homes that were seriously disadvantaged, dysfunctional, and disorganized. |
| **15. Ficks et al.(2013)** | Longitudinal | Georgia sample (N =1,676, mean age 8.6, 49% male)  Tennessee sample (N=4.038, mean age 11.7, 49% male) | Low birth weight.  monoamine oxidase A (MAOA) and catechol-O-methyltransferase (COMT) genes | ADHD, CD | Aim to better understand these associations in the context of potentially confounding genetic and environmental influences by examining phenotypic associations between birth weight and disruptive disorder symptoms both between families and within families in two independent twin samples. | We found negative associations between birth weight and inattentive, hyperactive-impulsive, and broad externalizing symptoms in both samples. Nonetheless, the overall magnitude of these associations was very small, contributing to less than 1% of the variance in these symptom dimensions. Within-family associations between birth weight and disruptive disorder symptoms did not differ for monozygotic and dizygotic twin pairs, suggesting that nonshared environmental influences rather than common genetic influences are responsible for these associations. |
| **16. Gibson et al. (2023)** | Longitudinal, retrospective cohort study | 4851 children born with placentas meeting criteria for, and 31,927 controls identified with normal placentas born during the same period. | Inflammation | ADHD, CD | Examine the association between FIRS and the risk for ADHD and CD in children and adolescents. | Children born to placentas meeting criteria for FIRS were more likely to be diagnosed with ADHD (OR = 1.27, CI 95% [1.07, 1.49]), conduct disorder (OR = 1.50, CI 95% [1.24, 1.81]), adjusting for maternal history of psychiatric disorders, intra-partem substance use, and prescriptions of anti-inflammatory drugs. |
| 1. **Grizenko & Pawliuk (1994)** | Case control | 50 disordered (mean age 9 yrs.) and 50 control preadolescents (aged 6–12 yrs.). | Learning difficulty, school failure, maternal stress | ADHD, CD | Explore the risk factors in the development of ADHD, conduct disorder and impulse control disorder. | Significant risk factors included learning difficulties, hyperactivity, perinatal complications, school failure, and a history of maternal stress. |
| 1. **Hill et al. (2000)** | Longitudinal retrospective case control | 50 children/adolescents (51.3% male). | Maternal drinking during pregnancy.  Maternal smoking during pregnancy. | CD | Examine the importance of prenatal exposure to cigarettes and alcohol in the etiology of childhood psychopathology. | Strong associations between prenatal maternal alcohol and cigarette use and the occurrence of CD. |
| **19. Huizink et al. (2007)** | Case-control twin study | Exposed (n = 232) and non-exposed Finnish twins (n = 572) were compared | Parental stress | ADHD, CD | To study the potential harmful effect of in utero exposure to the Chernobyl disaster in April 1986, and maternal anxiety associated with that exposure, on symptoms of behavior disorder observed at age 14. | In utero exposure to the Chernobyl disaster in 1986, and maternal anxiety presumably associated with that exposure shows an effect on depressive and attention deficit hyperactivity disorder symptoms in 14-year-olds. The effect is most prominent after exposure from second trimester onwards.  No differences in anxiety, conduct disorder or oppositional defiant disorder symptoms were found after prenatal stress exposure. |
| 1. **Humphreys et al. (2020)** | Longitudinal | At baseline (M age 22 months), 136 children from Bucharest, Romania, living in large institutions, were randomized into foster care (FCG) or to care as usual (CAUG). | Foster care placement/ adoption status | ADHD, CD | Examine the early psychological deprivation and its association with increased risk for psychopathology. | Ever-institutionalized children had higher rates of meeting criteria for any psychiatric disorder and higher symptom counts of externalizing, attention-deficit/hyperactivity, disorders compared to never-institutionalized children (PS < .05). Children in the CAUG had more than twice the rate of psychiatric disorders than children in the FCG (OR = 2.48, 95% CI [1.12, 5.48]). Furthermore, children in foster care who remained in their original placement did not significantly differ in their rates of psychiatric disorders compared to never-institutionalized children. |
| 1. **Knopik et al. (2005)** | Female twin study, longitudinal | 1091 monozygotic and 845 dizygotic twin pairs obtained from the Missouri Adolescent Female twin study. | Maternal drinking during pregnancy.  Maternal smoking during pregnancy. | ADHD | Examine the role of childhood maltreatment in children’s development. | ADHD was more likely to be diagnosed in girls whose mothers or fathers were alcohol dependent, whose mothers reported heavy alcohol use during pregnancy, and in those with low birthweight. |
| **22. Lahey et al. (1989)** | Cross-sectional | The biological mothers of 100 outpatient children aged 6-13 years completed the Minnesota Multiphasic Personality Inventory (MMPI). Child subjects ranged in age from 6 years, 4 months to 13 years, 6 months | Parental stress, maternal personality disorders. | ADHD, CD | Aim to explore the connection between maternal personality characteristics and the disruptive behavior disorders (such as conduct disorder and attention deficit/hyperactivity disorder) diagnosed in their children | These results indicate that CD is linked to maternal personality disorder and stress but ADD/H (ADHD) is not. |
| **23. Lambert (1988)** | Longitudinal | The longitudinal data contains the records of 367 subjects among which were 166 who were diagnosed and treated as hyperactive during childhood, 74 behavior controls who had either or both symptoms and behavior measures of hyperactivity but who were never considered to be hyperactive nor treated for it, and 127 subjects who were in the same schools as the hyperactive and behavior controls but who were asymptomatic as children. | Familial, social, and cognitive factors | ADHD, CD | Examine early life contributions of psychological characteristics, family environments, social relationships, cognitive and academic status, and school behavior, along with hyperactivity, in explaining each of several outcomes. | Regardless of the definition used, hyperactive children had significantly poorer educational outcomes and a greater extent of conduct disorders than their age peer controls. Regression analyses supported the inference that early biological factors, as well as the child's early health and temperament, predispose for adolescent mental health outcomes of depression, aggressive and nonaggressive conduct disorders, and hospitalization for psychological treatment. Familial, social, and cognitive factors had greater contributory potential in explaining educational outcomes, substance use, and conduct problems. |
| **24.Langley et al. (2007)** | Clinical cohort | 356 British Caucasian children aged between 6 and 16 years with a diagnosis of ADHD were referred to the study by Child and Adolescent Psychiatrists and Pediatricians in the Greater Manchester, South Wales, and Avon areas of the UK. | Low birthweight, maternal smoking in pregnancy and social class | ADHD, CD | Investigate possible effects of three indicators of environmental risk--maternal smoking during pregnancy, birth weight and social class--on comorbid CD. conduct disorder symptoms and inattentive and hyperactive-impulsive symptom severity | Greater hyperactive impulsive symptom severity was significantly associated with maternal smoking during pregnancy; Maternal smoking and social class predicted a diagnosis of CD. |
| 1. **Lorber & Egeland (2009)** | Longitudinal | Participants were 267 mothers and their firstborn children (54.7% male) from a high-risk urban population, recruited prenatally at the onset of an ongoing longitudinal study of development. | Poor quality early parenting | ADHD, CD | Examine whether the association of poor-quality infancy parenting and externalizing problems “rebounds” in adulthood. | Poor quality infancy parenting was associated with externalizing problems at kindergarten and first grade (mother report), as well as at 23 and 26 years (self-report). Infancy parenting was not significantly associated with either mothers' or youths' reports of externalizing problems at 16 years. These findings are consistent with the notion that poor quality infancy parenting is a risk factor for externalizing problems in developmental periods for which externalising behavior is most deviant. |
| **26.Mann et al. (2020)** | Longitudinal | Data came from the California Families Project, a longitudinal study of 674 Mexican-origin youth (50% female) and their parents. | Children’s personality traits | ADHD, CD | 1) tested whether a structure of common mental disorders within the hierarchical taxonomy of psychopathology was invariant from late childhood to adolescence in a sample of Mexican-origin youth, (2) examined the developmental course of psychopathology at different levels of the hierarchy, and (3) tested the degree to which changes in psychopathology were associated with changes in the Big Five personality domains. | Results of the present study indicate that a hierarchical model of common mental disorders extends to Mexican-origin youth and that developmental change in Big Five personality are related to developmental change in psychopathology. |
| **27.Meadows et al. (2007)** | Cohort study, longitudinal | 2,120 Families from the Fragile Families and Child Wellbeing Study. | Parental stress | ADHD | Examine the association between parental major depressive and generalized anxiety disorders and child behavior problems across family types: married, cohabiting, involved nonresident father, and noninvolved nonresident father. | Among 3-year-olds in all families, maternal anxiety/depression is associated with increased odds of anxious/depressed, attention deficit, and oppositional defiant disorders (N = 2,120). Paternal anxiety/depression has no significant association with these problem behaviors. |
| **28.Meehan et al. (2017)** | Longitudinal | 13,988 singletons/twins from The Avon Longitudinal Study of Parents and Children (ALSPAC) | Maternal stress, children’s specific personality traits | ADHD, CD | Aimed to identify low-anxiety (IC/ANX−) and high-anxiety (IC/ANX+) IC variants and compare these groups on (a) early risk exposures, (b) psychiatric symptoms from mid-childhood to early adolescence, and (c) school-based functioning | IC/ ANX+ youth showed the highest prenatal and postnatal levels of ADHD, CD and emotional difficulties, greatest discipline problems. |
| **29.Mick et al. (2002)** | Case control | 280 ADHD cases and 242 non-ADHD controls of both genders | Maternal smoking exposure, maternal alcohol use during pregnancy | ADHD | Address the putative association between ADHD and prenatal exposure to maternal cigarette smoking and alcohol attending to potential confounding by familial ADHD, maternal depression, conduct disorder, and indicators of social adversity in the environment. | ADHD cases were 2.1 times (95% confidence interval = 1.1-4.1; p = .02) more likely to have been exposed to cigarettes and 2.5 times (95% confidence interval = 1.1-5.5; p = .03) more likely to have been exposed to alcohol in utero than were the non-ADHD control subjects. Adjustment by familial psychopathology, Rutter’s indicators of social adversity, and comorbid conduct disorder did not account for the effect of prenatal exposure to alcohol or the products of cigarettes. |
| **30. Momany et al. (2017)** | Cross sectional | Participants included 915 children and adolescents ages 6 to 19 years (M 12.4, SD 4.3, 56.1% male). The sample included 431 singleton youth and 242 sibling pairs. | Low birth weight | ADHD, CD | Examine associations between BW and ADHD, ODD, and CD symptom dimensions as well as the extent to which such associations are moderated by child sex, while also controlling for confounding variables. | Significant interactions between sex and BW emerged across all analyses predicting ADHD and externalizing psychopathology, such that associations were stronger in males relative to females. Results remained when controlling for several confounds, including parental age, prenatal tobacco use, comorbid psychopathology, as well as other indicators of maternal and child health during the pre- and perinatal period. Both linear and quadratic associations emerged between BW and CD symptoms. |
| 1. **Montalvo-Ortiz et al. (2018)** | Case control | 44 subjects (22 with a DSM-5 diagnosis of intermittent explosive disorder and 22 comparable subjects without intermittent explosive disorder) | Inflammation | IED | Examine how do epigenetic changes, specifically DNA methylation patterns, contribute to the pathophysiology of IED, and what is the relationship between these epigenetic markers and the inflammatory, endocrine, and neuronal differentiation pathways implicated in IED | Inflammatory response as an important mechanism involved in intermittent explosive disorder Functional enrichment analysis revealed that genes mapped by these CpG sites are involved in the inflammatory/immune system, the endocrine system, and neuronal differentiation. |
| **32.Moore& Fombonne (1999)** | Longitudinal | Adopted children and adolescents under 18 years of age referred to the Maudsley Hospital, in South London, between 1983 and 1996 were considered for inclusion in the study. | Adoption | ADHD, CD | Explore: 1) the clinical and psychosocial profiles of adopted and nonadopted children attending a child psychiatric clinic, with reference to the relationship between disruptive disorders and adoptive status; 2) the relationship of age at adoption to childhood psychiatric disorder; and 3) the relationship between gender and psychiatric disorder within the adopted group. | Adopted boys and girls were both at increased risk of disruptive behavior, including conduct disorders and ADHD. Age at time of adoption was not related to psychopathology. |
| **33.Morgan et al. (2016)** | population-based, longitudinal | Children followed from kindergarten to eighth grade (N = 7,456) | Low SES family, maternal stress, childhood maltreatment, school failure | ADHD, CD | Identify which kindergarten children are simultaneously at risk of moderate or severe symptomatology in both attention-deficit/hyperactivity disorder (ADHD) and conduct disorder (CD) as adolescents. | Kindergarten children from low SES households, those raised by mothers with depressive symptoms or experiencing emotional problems or those who were punished by spanking were significantly more likely to later display severe levels of ADHD-CD symptomatology in eighth grade. Low academic achievement uniquely increased the risk of both moderate and severe symptomatology (adjusted OR range = 1.7 to 2.24). |
| **34.Nigg & Breslau (2007)** | Longitudinal | Data are from a longitudinal study of LBW (≤2,500 g) and normal birth weight children who were assessed at ages 6, 11, and 17. | Maternal Smoking during pregnancy | ADHD, CD | Examine prenatal smoking exposure and low birth weight as risk factors for attention-deficit/hyperactivity disorder (ADHD), and conduct disorder (CD) in a population-based longitudinal design from ages 6 to 17 years. | The association of prenatal smoking exposure with ADHD was highly confounded by family variables. In contrast, low birth weight independently predicted ADHD, even with family variables statistically controlled. The opposite pattern appeared for CD. Prenatal smoking exposure also predicted CD. |
| **35.Owens & Hinshaw (2016)** | Longitudinal | Data came from 140 participants in the Berkeley Girls with ADHD Longitudinal Study. | School failure, classroom misconduct | ADHD, CD | Test whether conduct problems predicted young adult functioning and psychiatric symptoms among women diagnosed with attention-deficit/hyperactivity disorder (ADHD) during childhood, in the context of 3 potential adolescent mediators: internalizing problems, peer rejection, and school failure and disciplinary problems. | Adolescent school failure and disciplinary problems mediated the relations between childhood conduct problems and both young adult functioning and externalizing problems; adolescent internalizing problems and peer conflict mediated the relation between childhood conduct problems and young adult internalizing problems. As is true for boys, childhood and adolescent conduct problems are associated with poor adult outcomes among girls with ADHD, with school failure and disciplinary problems, internalizing problems, and peer conflict functioning as mediators of these relations. |
| **36.Puhalla et al. (2020)** | Case control | 493 individuals (68% female; Age M = 26.65) either with (n = 265) or without (psychiatric control group; n = 228) IED | Childhood abuse | IED | Examine the relative contributions of childhood abuse and AUD to IED. | A history of childhood abuse may increase the chances of engaging in overall aggression and developing IED, which in turn may increase the association between AUD severity and intoxicated aggression. |
| **37.Sasaluxnanon & Kaewpornsawan (2005)** | Retrospective, hospital-based, case control study | 122 ADHD cases from the child and adolescent psychiatric outpatient unit at Siriraj Hospital, 119 non-ADHD from students who have been assessed from Child Behavior Checklist (CBCL) and Conners Teacher Rating Scale, they are identified as normal. | Low birth weight | ADHD | To study the factor of birth weight below 2,500 grams and ADHD and to identify the factors that are associated with ADHD. | The number of ADHD cases who had a birth weight below 2,500 grams was 3.6 times the number of control cases who had a birth weight below 2,500 grams. It was statistically significant (p = 0.03). Factors associated with ADHD were pregnancy complication (p value < 0.05, OR = 4.17, 95%CI [1.66, 14.02]), emotional distress during pregnancy (p value <0.05, OR = 2.99, 95%CI [1.43, 5.40]), postnatal complication (p value < 0.05, OR = 3.26, 95%CI[1.56, 6.41] and a family history of ADHD (p value < 0.05, OR = 3.6, 95%CI [1.65, 8.11]. |
| **38. Scott et al. (2012)** | Longitudinal | The EPT/ELBW group consisted of children with <28 weeks GA and/or <1000 g birth weight born between 2001 and 2003 and treated in the neonatal intensive care unit of Rainbow Babies & Children’s Hospital in Cleveland, Ohio. Of the 198 children without congenital abnormalities or infections who survived, 148 (75%) were recruited for this study. | Low birth weight | ADHD, CD | The primary aim of this study was to assess the types and extent of behavior disorders in a 2001–2003 birth cohort of EPT/ELBW children during this first year in school compared with NBW classmate controls.  A secondary aim was to determine if impairments in tests of global cognitive ability or executive function increase the risk for behavior disorders in EPT/ELBW children at school entry. | Rates of ADHD combined on psychiatric interview were about twice as high for the LBW group than for the NBW group, OR (95% CI) =2.50 (1.34, 4.68), p=.004. The LBW group also had much higher rates of teacher-identified disorders in attention, behavior self- regulation, and social functioning, with odds ratios (95% confidence intervals) ranging from 3.35 (1.64, 6.83) to 18.03 (4.12, 78.94), all p’s<.01. ADHD and impaired behavior self-regulation were associated with deficits on tests of executive function. |
| **39.Shaw et al. (2001)** | Retrospective, cross-sectional | N =1145 adult individuals participated. | childhood adversaries | IED | Examine the underlying childhood environment of those with IED, particularly familial and school-related factors. | Significant patterns emerged specific to IED for not being raised by both parents, greater physical aggression to participant, and greater degree of fighting with peers by age ten; Results suggest the prevalence childhood adversaries may be linked with IED; the childhood environment of those with IED likely is substantially more tumultuous than individuals with or without other psychiatric disorders. |
| **40. Shevidi et al. (2023)** | Cross-sectional | Eleven-hundred-forty-five (n = 1145) adult individuals participated in this study. | participant adoption status, intrafamilial physical aggression witnessed and experienced, frequency of contact with non-primary caregiver | IED | Investigate the parent marital status, participant adoption status, intrafamilial physical aggression witnessed and experienced, frequency of contact with non-primary caregiver (in cases of parental separation), syndromal and personality psychopathology, neurodevelopmental and learning difficulties, behavioral issues at school, peer relationships, and juvenile legal concerns of participants with IED. | Significant patterns emerged specific to IED for not being raised by both parents, greater physical aggression to participant, and greater degree of fighting with peers by age ten. |
| **41.Stein et al. (2006)** | Population-based, longitudinal | 7817 children, 0 to 12 years of age, from the sample child file of the 2002 National Health Interview Survey | Low birth weight | ADHD | Examine whether moderately low birth weight children were at greater risk for health problems than normal birth weight children in a nationally representative sample of US children. | Moderately low birth weight children were significantly more likely than normal birth weight children to be identified as having a special health care need, having a chronic condition, having a learning disability, and having ADHD. They were not more likely to have a hospitalization in the past year. |
| **42.Sullivan et al (1995)** | Cross-sectional | A total of 1498 people were interviewed, and the overall participation rate was 70 %. | Adoption | CD | Examine whether adoption is a risk factor for mental disorders in adults. | In comparison to individuals raised by both biological parents, adoption was strongly associated with a history of childhood conduct disorder |
| **43.Wakschlag et al. (2006)** | Clinical cohort, longitudinal | Boys from the youngest cohort of the Pittsburgh Youth Study (N = 448). | Maternal smoking exposure during pregnancy | ADHD | Use a developmental framework to examine the association of exposure with (1) ADHD in young boys and (2) the pattern of delinquent behavior at adolescence. | Exposed boys were significantly more likely to (1) develop oppositional defiant disorder and comorbid oppositional defiant disorder-attention-deficit/hyperactivity disorder but not attention-deficit/hyperactivity disorder alone and (2) to have an earlier onset of significant delinquent behavior. |
| **44.Weissman et al. (1999)** | Longitudinal | Fifty offspring of mothers who reported smoking at least 10 cigarettes almost daily during pregnancy and 97 offspring of mothers who reported never smoking during pregnancy were studied. | Maternal smoking exposure during pregnancy | ADHD, CD | To extend findings from several independent reports of an association between maternal smoking during pregnancy and attention-deficit hyperactivity disorder, conduct disorder, and substance abuse in the offspring. | There was a greater than 4-fold increased risk of prepubertal-onset conduct disorder in boys and a greater than 5-fold increased risk of adolescent-onset drug dependence in girls whose mothers smoked 10 or more cigarettes almost daily during pregnancy. |
